# Potential prognostic value of CSF-targeted proteomics across the Alzheimer’s disease continuum

**DOI:** 10.1186/s12877-024-05104-z

**Published:** 2024-06-06

**Authors:** Bingdong Xu, Yitong Ling, Leiyuan Liu, Yujun Liu, Yingze Lin, Jun Lyu, Yusheng Zhang

**Affiliations:** 1 Department of Neurology, The First Affiliated Hospital of Jinan University, No. 613, Huangpu Avenue West, Guangzhou, Guangdong 510632 P.R. China; 2 Department of Clinical Research, The First Affiliated Hospital of Jinan University, Guangzhou, China; 3 Guangdong Provincial Key Laboratory of Traditional Chinese Medicine Informatization, Guangzhou, China

**Keywords:** Alzheimer’s disease, Proteomics, Biomarkers, Prognosis, Cognitive function

## Abstract

**Background:**

Core biomarkers for Alzheimer’s disease (AD), such as Aβ42 and tau, have demonstrated high prognostic accuracy but do not fully capture the complex pathophysiology of AD. In this study, our objective was to identify novel cerebrospinal fluid (CSF) biomarkers using proteomics across the entire AD continuum to predict conversion to AD and explore their involvement in AD pathogenesis.

**Methods:**

A cohort of 186 cognitively normal (CN), 127 subjective memory complaint (SMC), 79 early mild cognitive impairment (EMCI), 249 late MCI (LMCI), and 132 AD individuals was analyzed, with a follow-up period of over 3 years for non-AD participants. CSF 65 peptides, as well as hippocampal and entorhinal volumes were analyzed, and cognitive function was evaluated using the 13-item cognitive subscale of the Alzheimer’s Disease Assessment Scale (ADAS-Cog 13). Cox proportional hazards models and mediation analysis were performed to investigate associations and causal relationships.

**Results:**

During the follow-up, approximately one-fourth (146/580) of the non-AD participants progressed to AD. After adjusting for baseline diagnosis (CN to LMCI) and other variables, multivariable Cox regression analysis identified three peptides (VAELEDEK, VSFELFADK, and VVSSIEQK) as significant predictors of conversion to AD. Incorporating these three peptides into the initial model significantly improved the C-statistic from 0.82 to 0.85 for predicting AD conversion, surpassing the predictive ability of Aβ42 and P-tau. Moreover, hippocampal and entorhinal volumes mediated 30.3–53.8% of the association between the three peptides and ADAS-Cog 13 scores.

**Conclusions:**

These findings underscore the potential of these three peptides as robust prognostic biomarker candidates for AD conversion across the entire AD continuum, with a mechanism involving the mediation of hippocampal and entorhinal volumes.

**Supplementary Information:**

The online version contains supplementary material available at 10.1186/s12877-024-05104-z.

## Introduction

Alzheimer’s disease (AD) is a debilitating and progressive neurodegenerative disorder that results in severe cognitive and behavioral impairments [1]. AD follows a continuum, starting from an asymptomatic phase, to mild cognitive impairment (MCI), and eventually progressing to the final stage of dementia [2]. It is characterized by the degeneration of brain tissue, particularly in the hippocampus [3] and entorhinal cortex [4], which are closely associated with initial progressive memory loss. Currently, most research focuses on the β-amyloid (Aβ) and tau proteins, which are major components of amyloid plaques and neurofibrillary tangles. Cerebrospinal fluid (CSF) Aβ and tau have demonstrated high diagnostic and predictive accuracy for AD [5, 6]. However, the clinical failures of some drugs targeting Aβ clearance suggest that these biomarkers do not fully capture the complex and multifactorial pathogenic processes occurring in different stages of AD [1, 7]. In reality, although Aβ deposition and tau-related hyperphosphorylation are core mechanisms of AD pathogenesis, other molecular changes have been reported throughout the AD continuum [8, 9].

Proteomics, utilizing liquid chromatography-tandem mass spectrometry (LC-MS/MS), is an unbiased and powerful technique that enables the identification and quantification of proteins based on peptide-level amino acid sequence information. This technique has led to the discovery of numerous proteins capable of identifying AD [10], unveiling the mitochondrial characteristics of AD [11], and revealing changes in energy metabolism associated with glial cell activation [12]. However, most studies primarily focus on identifying AD and unraveling its pathogenic mechanisms, with limited research on using proteomics to predict the conversion from non-AD to AD. Moreover, it may be more effective and rational to utilize differential proteins spanning all stages of the AD disease spectrum for predicting the conversion to AD. To address this, the Alzheimer’s Disease Neuroimaging Initiative (ADNI) has undertaken a high-throughput detection and quantification of proteins in CSF samples using LC-MS/MS to identify differential proteins across the AD continuum [13–15]. Unlike previous studies, the ADNI study emphasizes the expression of AD-associated proteins across all stages, ranging from cognitively normal (CN), subjective memory complaint (SMC), early MCI (EMCI), late MCI (LMCI), and progressing to AD. This comprehensive approach allows for the observation of dynamic proteomic changes throughout the entire AD development process.

In the ADNI study, a comprehensive analysis was conducted on the entire AD continuum, measuring sixty-five peptides. Our approach involved initially screening for differential peptides that are common across the other four stages in comparison to AD. Subsequently, the accuracy of predicting the conversion to AD in non-AD individuals by these differential peptides was assessed. Furthermore, the potential mechanisms through which these differential peptides impact cognitive function were investigated. This study makes a significant contribution to understanding the predictive value of shared differential peptides in the AD continuum and sheds light on their involvement in the early stages of AD.

## Methods

### Participants

The data used in this study were obtained from the ADNI database, accessible at adni.loni.usc.edu. ADNI was initiated in 2003 under the leadership of Principal Investigator Michael W. Weiner, MD, as a collaborative effort between the public and private sectors. The database comprises a wide range of data, including CSF biomarkers, magnetic resonance imaging, positron emission tomography, and neuropsychological assessments. These data were collected from individuals across various cognitive states, including those with CN, SMC, EMCI, LMCI, and AD. For further details, please refer to www.adni-info.org. All research sites participating in ADNI have received approval from their respective local Institutional Review Boards, and all participants or their authorized representatives have provided written informed consent. The ethical approval for ADNI 1, GO, 2, and 3 can be found on ClinicalTrials.gov with the following identifiers: NCT00106899, NCT01078636, NCT01231971, and NCT02854033, respectively.

In this study, A total of 773 participants with complete CSF proteomics data were initially identified from the “Emory University CSF Targeted MS SET2 [ADNI1, GO, 2, 3].csv” file in the ADNI database. These participants consisted of 186 CN, 127 SMC, 79 EMCI, 249 LMCI, and 132 AD. The diagnostic criteria for each group have been previously described in detail [16, 17]. In summary, CN individuals were defined as having Mini-Mental State Examination scores between 24 and 30, a Clinical Dementia Rating of 0, no depression, no mild cognitive impairment, and no dementia. SMC individuals were defined as having a score of ≥ 16 on the first 12 items of the Cognitive Change Index. LMCI individuals adjusted the scores for measuring objective memory loss in combination with the level of education, compared to EMCI individuals. The diagnosis of AD was based on the National Institute of Neurological and Communicative Disorders and Stroke/Alzheimer’s Disease and Related Disorders Association criteria. Subsequently, baseline demographic information, CSF biomarkers, hippocampal and entorhinal volumes, and cognitive function data were extracted from the “ADNIMERGE-Key ADNI tables merged into one table.csv” and “UPENN CSF Biomarker Master [ADNI1, GO, 2, 3].csv” files. Additionally, follow-up data on the progression from non-AD to AD were also obtained from the “ADNIMERGE-Key ADNI tables merged into one table.csv”.

### Measurement of CSF-targeted proteomics

Baseline proteomics data was collected from participants enrolled in the ADNI cohort. The targeted proteomics analysis of CSF samples was conducted by the Department of Neurology, Emory University School of Medicine, using mass spectrometry. The methods employed were previously described in the study and involved the analysis of peptide ratios for sixty-five peptides associated with forty-seven proteins [18]. In brief, the CSF samples underwent a series of processing steps, including reduction, alkylation, denaturation, and enzymatic digestion using Lys-C and trypsin. The resulting peptides were then analyzed using a standard flow Agilent 1290 Infinity II liquid chromatography system coupled with a Thermo Fisher Scientific TSQ Altis Triple Quadrupole mass spectrometer. Isotopically labeled peptide standards were added for relative quantification, and the total area ratios for the targeted peptides were reported. For more detailed information on the protein names corresponding to the peptides and the specific detection procedures, please refer to the following link: https://ida.loni.usc.edu/download/files/study/f28033a7-1734-437f-84f9-c4fd2ae2460f/file/adni/ADNI_MethodsReport_EmoryRevised_20221019.pdf.

### Measurement of CSF Aβ42, tau, and P-tau

The concentrations of baseline CSF Aβ42, tau, and P-tau in samples from ADNI participants were measured using the multiplex xMAP Luminex platform (Luminex Corp, Austin, TX, USA) and the INNOBIA AlzBio3 kit (Fujirebio, Ghent, Belgium). The CSF samples were analyzed at the laboratory of the University of Pennsylvania.

### Measurement of hippocampal and entorhinal volumes

Baseline head images of the participants were acquired using T1-weighted sagittal 3D magnetization-prepared rapid gradient-echo sequences on a 3 Tesla MRI scanner. The images were then processed using FreeSurfer 5.1 to extract the hippocampal and entorhinal volume. FreeSurfer is a widely used software package for automated segmentation and volumetric measurements of brain structures from images.

### Neuropsychological assessment

Despite the presence of multiple cognitive assessment scales in the ADNI study, the baseline cognitive function of the participants was evaluated using the 13-item cognitive subscale of the Alzheimer’s Disease Assessment Scale (ADAS-Cog 13), considering the completeness of scoring data and the comprehensiveness of cognitive function assessment. This assessment measures various cognitive domains, including memory, attention, language, orientation, and executive function. The total score of ADAS-Cog 13 ranges from 0 to 85, with higher scores indicating more severe cognitive impairment.

### Endpoints

Follow-up was conducted on participants who were diagnosed with CN, SMC, EMCI, and LMCI at baseline. The endpoint event was defined as the conversion to AD. For each participant, the number of months from the first diagnosis of AD was extracted from the database. If a participant was not diagnosed with AD at their last follow-up, the number of months from baseline to the last follow-up was recorded. This allowed us to track the progression of the participants over time and assess the rate of conversion to AD.

### Statistical analysis

All statistical analyses were performed using SPSS software (version 26.0; IBM SPSS) and R (version 4.2.3) with a significance level of *P* < 0.05 on both sides, unless otherwise stated. Descriptive data were presented as mean and standard deviation, median and interquartile range, or numbers and percentages. Differences in numerical variables among multiple groups were assessed using one-way ANOVA or Kruskal-Wallis test, depending on the normality of the data. Multiple imputation techniques were used to fill missing values. The details regarding the missing covariates can be found in Supplementary Table 1.

To assess the differential expression of sixty-five peptides, the Mann-Whitney U test was used and *P* values were corrected for multiple testing using the false discovery rate method of Benjamini-Hochberg (*P* < 0.05). This analysis identified peptides with significantly altered abundance levels between different groups, and volcano plots were generated using GraphPad Prism 8.0. Receiver operator characteristic (ROC) curves were employed to evaluate the diagnostic value of peptides, as well as CSF Aβ42, tau, and P-tau among different groups. Positive predictive value (PPV) and negative predictive value (NPV) were calculated based on the ratio of cases in the positive and negative groups to reflect the prevalence of the disease. Additionally, DeLong’s test was used to compare the diagnostic accuracy of these biomarkers among different groups.

Univariable Cox proportional hazards model was used to evaluate the association between those variables and AD. Considering the limited number of instances for the outcome variable, variables with a p-value less than 0.05 were included in the multivariable Cox proportional hazards models. Hippocampal and entorhinal volumes, as well as ADAS-Cog 13, were not included in the analysis as they were considered outcomes of disease progression. CSF peptides and Aβ42, tau, and P-tau levels were log10 transformed for standardization. To validate the robustness of our findings, participants with incomplete data were excluded, and the univariable and multivariable Cox regression analysis were conducted using complete datasets. Kaplan-Meier survival analysis was performed using the log-rank test to compare the differences in survival rates among CN, SMC, EMCI, and LMCI. The C-statistic was used to assess the incremental discriminative value of differential peptides in predicting the conversion to AD.

Furthermore, mediation analysis was conducted using the PROCESS macro, version 3.5, to investigate the indirect effects of peptides on cognitive function through hippocampal and entorhinal volumes. Total, direct, and indirect effects were calculated using bootstrapping with 5000 iterations. The analysis included peptides that showed significant differences in the Cox proportional hazards analysis and were log10 transformed for standardization.

To validate the robustness of our findings, we conducted sensitivity analyses. Participants with incomplete data were excluded, and the differential expression of sixty-five peptides, the univariable Cox regression, and multivariable Cox regression analyses were conducted using complete datasets.

## Results

### Identification of AD-associated peptides

**Fig. 1:**
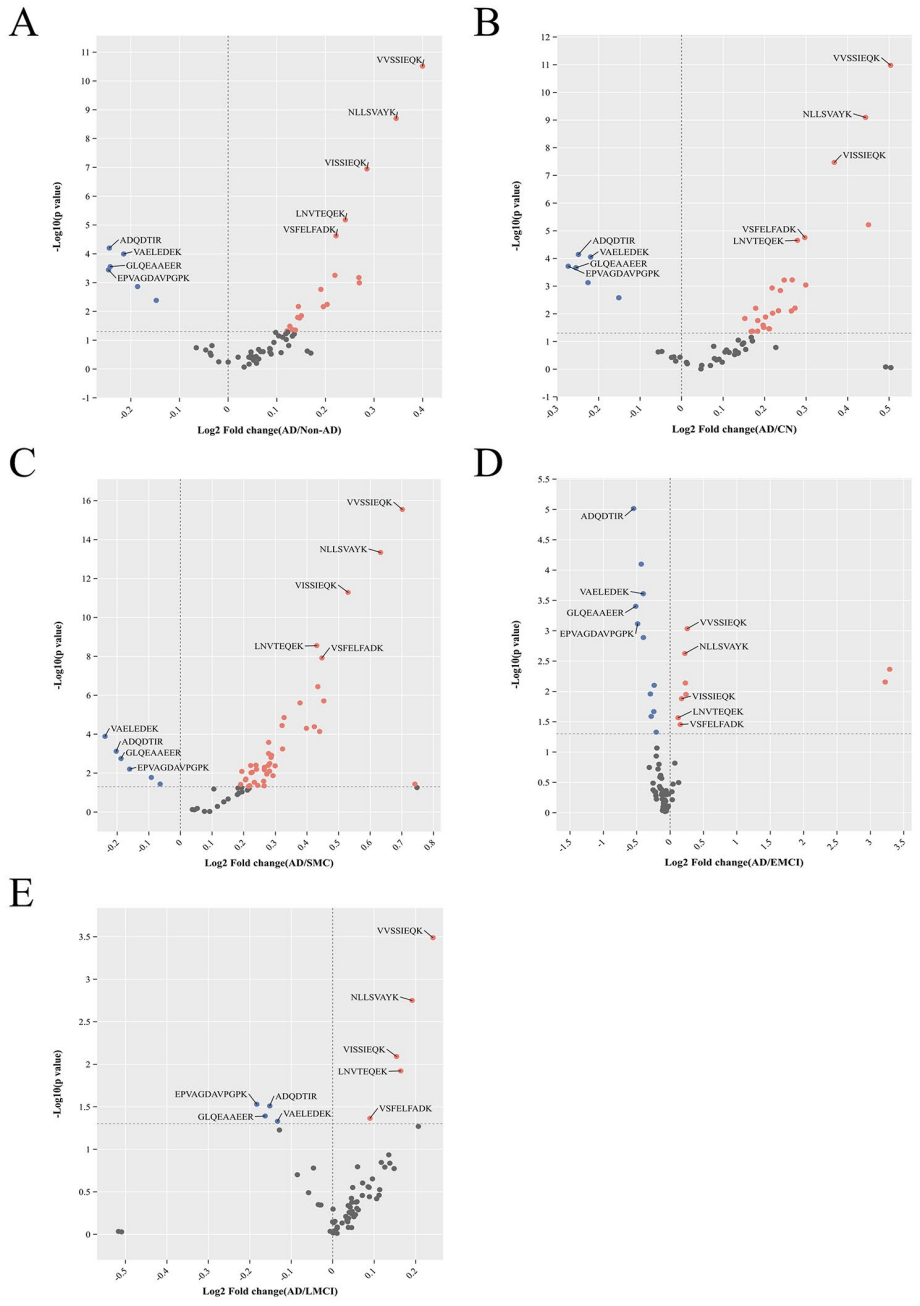
Volcano plots of differential protein expression between every two groups. Blue dots represented peptides with low expression, red dots represented peptides with high expression, and gray dots represented peptides with no differential expression. Nine peptides that showed differential expression were presented across the AD continuum. Abbreviations: CN, cognitively normal; SMC, subjective memory complaint; EMCI, early mild cognitive impairment; LMCI, late mild cognitive impairment; AD, Alzheimer’s disease

LC-MS/MS was utilized to measure the levels of sixty-five peptides in 773 subjects. The distribution of participants among different groups was as follows: 186 in the CN group, 127 in the SMC group, 79 in the EMCI group, 249 in the LMCI group, and 132 in the AD group. Figure 1 illustrates the differences in peptide abundance between the various non-AD groups and the AD group, displaying nine differential peptides across the AD continuum. As the severity of cognitive impairment increased, the number of differential peptides decreased (Fig. 1B, D, E). Interestingly, the SMC group had the highest number of differential peptides compared to the AD group (Fig. 1C).

The role different peptides may play in different stages of AD development can vary. To achieve the best predictive effect with the minimum number of differential peptides, we selected nine peptides that showed differential expression in all different stages indicated in Fig. 1 for further analysis. Among these peptides, four peptides showed relatively downregulated expression: ADQDTIR, EPVAGDAVPGPK, GLQEAAEER, and VAELEDEK. These peptides correspond to the proteins neuronal pentraxin receptor (NPTXR), neurosecretory protein VGF (VGF), VGF, and neuronal pentraxin-2 (NPTX2), respectively. On the other hand, five peptides showed relatively upregulated expression: LNVTEQEK, NLLSVAYK, VISSIEQK, VSFELFADK, and VVSSIEQK. These peptides correspond to the proteins Enolase 1 (ENO1), 14-3-3 beta/alpha, 14-3-3 beta/alpha, peptidyl-prolyl cis-trans isomerase (PPIase) A, and 14-3-3 protein zeta/delta, respectively. Additionally, in Aβ-positive participants, there were five common differential peptides (VAELEDEK, VSFELFADK, VVSSIEQK, NLLSVAYK, and LNVTEQEK), which were also part of the previously identified nine differential peptides. This indicated differences in certain peptides between Aβ-positive and Aβ-negative participants. Table 1 illustrates significant differences among groups in demographic characteristics and AD biomarkers.

**Table 1:** Demographics and clinical characteristics (*n* = 773)

Characteristics	CN (*n* = 186)	SMC (*n* = 127)	EMCI (*n* = 79)	LMCI (*n* = 249)	AD (*n* = 132)	*P* value
Age, years	73.0 ± 6.2	71.0 ± 6.3	71.4 ± 7.5	73.7 ± 7.6	73.8 ± 8.4	0.003^**^
Female, n (%)	107 (57.5)	82 (64.6)	36 (45.6)	83 (33.3)	53 (40.2)	< 0.001^***^
Hisp/Latino, n (%)	6 (3.2)	5 (3.9)	4 (5.1)	5 (2.0)	2 (1.5)	0.437
Education, years	16 (14–18)	17 (16–18)	16 (13–18)	16 (14–18)	16 (13–18)	< 0.001^***^
Married status, n (%)	136 (73.1)	92 (72.4)	69 (87.3)	208 (83.5)	114 (86.4)	0.001^**^
ApoE ε4, n (%)	44 (23.7)	51 (40.2)	32 (40.5)	132 (53.0)	94 (71.2)	< 0.001^***^
Aβ positivity, n (%)	134 (72.0)	49 (38.5)	44 (55.6)	230 (92.3)	130 (98.4)	< 0.001^***^
CSF Aβ42 (pg/ml)	261.5 (202.7–1120.7)	1095.0 (811.9–1675.0)	827.0 (234.0–1406.0)	172.0 (132.5 -287.5)	149.0 (129.0–335.0)	< 0.001^***^
CSF tau (pg/ml)	93.0 (58.0–182.9)	215.4 (169.4- 271.7)	213.6 (128.0–281.5)	105.0 (69.0–176.5)	139.5 (90.5–219.5)	< 0.001^***^
CSF P-tau (pg/ml)	19.0 (15.0–25.0)	19.0 (14.5–25.4)	21.9 (17.3–38.2)	29.7 (19.0–43.5)	35.0 (27.8–47.3)	< 0.001^***^

Note: Values are expressed as mean ± SD, median (interquartile range), or frequency (%)

Abbreviations: CN, cognitively normal; SMC, subjective memory complaint; EMCI, early mild cognitive impairment; LMCI, late mild cognitive impairment; AD, Alzheimer’s disease; ApoE, apolipoprotein E; CSF, cerebrospinal fluid; Aβ, β-amyloid

*P < 0.05; **P < 0.01; ***P < 0.001

### Longitudinal analysis: newly developed AD events during follow-up

Follow-up was conducted on the four non-AD groups (CN, SMC, EMCI, LMCI) to document the time of conversion to AD. Among the 641 non-AD participants, 61 were lost to follow-up, leading to a final analysis of 580 participants. The median follow-up time was 36 months (range: 24 to 48). Over the follow-up period, a total of 146 participants converted to AD.

The survival analysis using the Cox regression model showed that gender, married status, ApoE ε4, baseline diagnosis, Log Aβ42, Log P-tau, low-expressed peptides (Log ADQDTIR, Log EPVAGDAVPGPK, Log GLQEAAEER and Log VAELEDEK), and high-expressed peptides (Log LNVTEQEK, Log NLLSVAYK, Log VISSIEQK, Log VSFELFADK and Log VVSSIEQK) were univariable predictors of the endpoint event (conversion to AD). Furthermore, the multivariable Cox regression analysis revealed that Log VAELEDEK (hazard ratio [HR] = 0.05; 95% CI = 0.01–0.44; *P* = 0.007), Log VSFELFADK (HR = 0.01; 95% CI = 0.00–0.09; *P* = 0.001), and Log VVSSIEQK (HR = 38.93; 95% CI = 2.62–577.48; *P* = 0.008) remained significant predictors of the conversion to AD, along with gender, baseline diagnosis, Log Aβ42, and Log P-tau (Table 2). Furthermore, the initial combined model of Aβ42 and P-tau exhibited a C-statistic of 0.82 in predicting the progression to AD. However, the incorporation of these three additional peptides resulted in an improved C-statistic of 0.85 (*P* < 0.001).

**Table 2:** Univariable and multivariable Cox regression analysis for conversion to AD as the outcome measure in non-AD patients (*n* = 580)

Parameter	Univariable analysis HR (95% CI)	*P* value	Multivariable analysis HR (95% CI)	*P* value
Age	1.02 (1.00–1.05)	0.077		
Female	0.69 (0.49–0.96)	0.029^*^	1.35 (0.91–2.00)	0.140
Education	0.95 (0.90–1.01)	0.075		
Married status	0.49 (0.29–0.82)	0.007^**^	0.70 (0.40–1.23)	0.211
Hisp/Latino	0.56 (0.14–2.26)	0.413		
ApoE ε4	2.68 (1.92–3.74)	< 0.001^***^	1.06 (0.71–1.56)	0.788
**Baseline diagnosis**				
CN	Reference			
SMC	0.21 (0.03–1.62)	0.135	1.15 (0.14–9.70)	0.897
EMCI	3.80 (1.77–8.13)	0.001^***^	5.44 (2.40–12.29)	< 0.001^***^
LMCI	13.17 (7.47–23.22)	< 0.001^***^	10.06 (5.33–18.98)	< 0.001^***^
**AD biomarkers**				
Log Aβ42	0.03 (0.01–0.07)	< 0.001^***^	0.23 (0.10–0.56)	0.001^**^
Log Tau	1.11 (0.63–1.96)	0.728		
Log P-tau	40.73 (19.39–85.53)	< 0.001^***^	4.72 (1.64–13.63)	0.004^**^
**Underexpressed peptides**				
Log ADQDTIR	0.19 (0.08–0.44)	< 0.001^***^	0.17 (0.01–1.99)	0.156
Log EPVAGDAVPGPK	0.26 (0.13–0.54)	< 0.001^***^	0.24 (0.01–10.82)	0.461
Log GLQEAAEER	0.24 (0.11–0.51)	< 0.001^***^	0.76 (0.02–34.22)	0.890
Log VAELEDEK	0.14 (0.05–0.37)	< 0.001^***^	0.05 (0.01–0.44)	0.007^**^
**Overexpressed peptides**				
Log LNVTEQEK	5.44 (1.97–15.07)	0.001^**^	4.60 (0.44–48.34)	0.203
Log NLLSVAYK	7.35 (3.25–16.65)	< 0.001^***^	8.33 (0.11–632.31)	0.337
Log VISSIEQK	8.16 (3.24–20.58)	< 0.001^***^	10.27(0.13–822.38)	0.298
Log VSFELFADK	3.72 (1.62–8.52)	0.002^**^	0.01 (0.00–0.09)	< 0.001^***^
Log VVSSIEQK	9.85 (4.49–21.58)	< 0.001^***^	38.93 (2.62–577.48)	0.008^**^

Note: AD cerebrospinal fluid biomarkers and peptides were normalized by log_10_ transformation

Abbreviations: CN, cognitively normal; SMC, subjective memory complaint; EMCI, early mild cognitive impairment; LMCI, late mild cognitive impairment; AD, Alzheimer’s disease; Aβ, β-amyloid; HR, hazard ratios; CI, confidence interval

*P < 0.05; **P < 0.01; ***P < 0.001

**Fig. 2:**
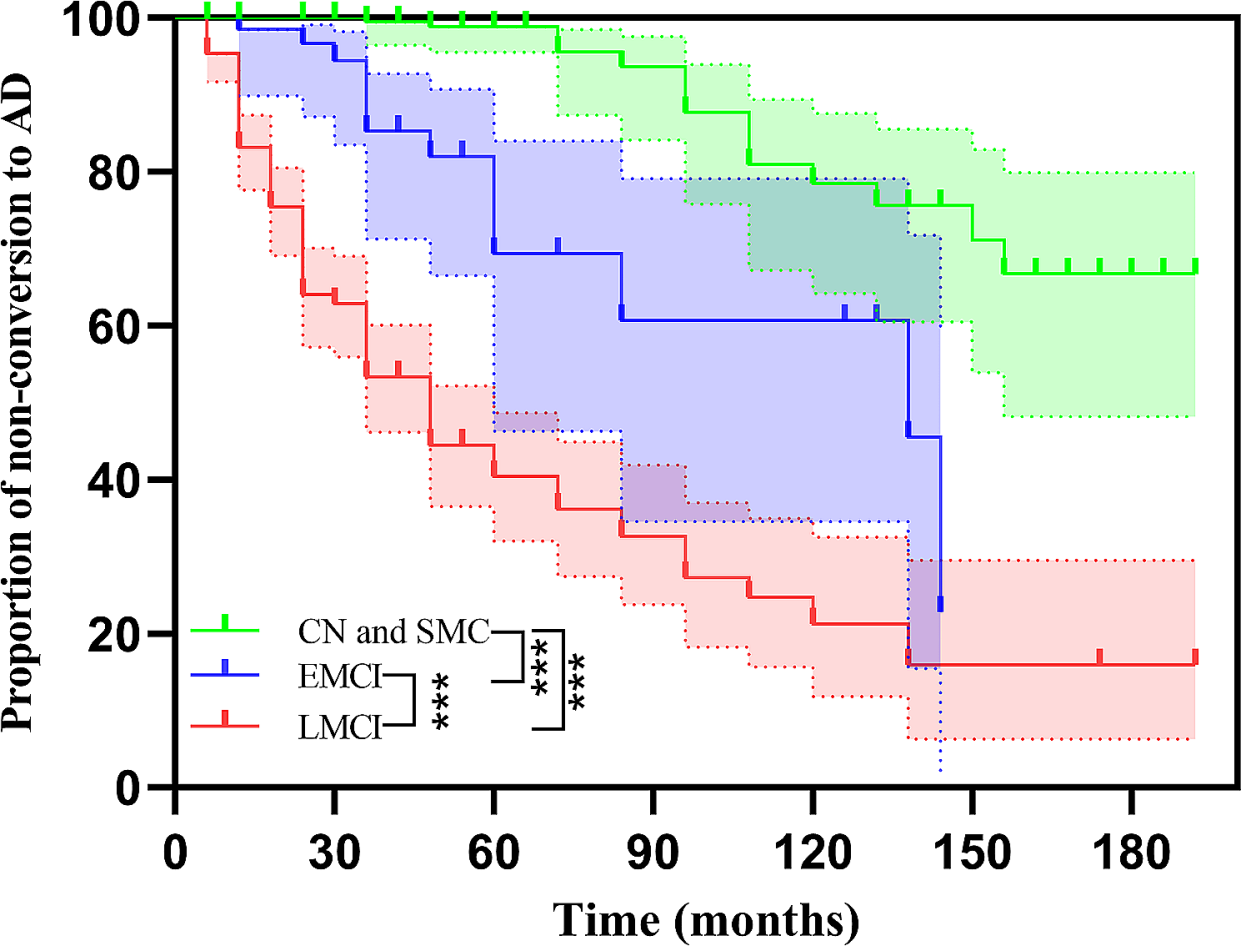
Kaplan-Meier survival curves of non-conversion to AD. There were statistically significant differences between the CN and SMC vs. EMCI, CN and SMC vs. LMCI, and EMCI vs. LMCI groups. Abbreviations: CN, cognitively normal; SMC, subjective memory complaint; EMCI, early mild cognitive impairment; LMCI, late mild cognitive impairment; AD, Alzheimer’s disease. ***P < 0.001

In the multivariable Cox regression model, with CN as the reference group, it was observed that as the severity of baseline cognitive impairment increased from EMCI to LMCI, the risk of conversion to AD also increased. Specifically, the risk of conversion to AD was 5.44 times higher for individuals with EMCI and 10.06 times higher for individuals with LMCI compared to CN. However, individuals with SMC did not show a significant increase in the risk of progressing to AD compared to CN. Considering that there was no significant difference in the progression to AD between CN and SMC, in the Kaplan-Meier survival analysis, CN and SMC were combined into one group. The results showed that baseline diagnosis was a useful predictor for the progression to AD, with the risk of developing AD ranked highest for baseline diagnosis of LMCI, followed by EMCI, and CN combined with SMC (*P*-all < 0.001, Fig. 2).

### Association between targeted peptides and AD biomarkers

We analyzed whether these peptides correlated with other CSF markers of AD pathology in this cohort. Linear regression model analysis of CSF Aβ42, P-tau, and targeted peptides, controlling for age, gender, education, and ApoE ε4 as possible confounders, is shown in Table 3 for 773 subjects. CSF Aβ42 showed significant positive correlations with VAELEDEK and VSFELFADK, but significant negative correlations with VVSSIEQK (*P*-all < 0.001). Additionally, CSF P-tau exhibited a significant positive correlation with VVSSIEQK, while CSF Aβ42 was significantly negatively correlated with VSFELFADK (*P*-all < 0.001).

**Table 3:** Multivariable linear regression between cerebrospinal fluid AD biomarkers and targeted peptides (*n* = 773)

Parameter	Coefficient	95% CI	*P* value
**Aβ42**			
Log VAELEDEK	0.695	0.506–0.883	< 0.001^***^
Log VSFELFADK	0.322	0.055–0.589	< 0.001^***^
Log VVSSIEQK	-0.944	-1.221 - -0.667	< 0.001^***^
**P-tau**			
Log VAELEDEK	-0.004	-0.094–0.086	0.925
Log VSFELFADK	-0.291	-0.418 - -0.164	< 0.001^***^
Log VVSSIEQK	0.823	0.961 − 0.955	< 0.001^***^
**ADAS-Cog 13**			
Log VAELEDEK	-0.591	-0.714–0.467	< 0.001^***^
Log VSFELFADK	-0.219	-0.392 - -0.045	0.014^*^
Log VVSSIEQK	0.785	0.604–0.965	< 0.001^***^

Note: AD cerebrospinal fluid biomarkers and peptides were normalized by log_10_ transformation. Age,

gender, education, and ApoE ε4 were controlled as possible confounders in multivariable linear regression

Abbreviations: AD, Alzheimer’s disease; Aβ, β-amyloid; ADAS-Cog 13, 13-item cognitive subscale of the Alzheimer’s Disease Assessment Scale; HR, hazard ratios; CI, confidence interval

*P < 0.05; **P < 0.01; ***P < 0.001

### Diagnostic value of targeted peptides in group differentiation

Utilizing ROC curve analysis, we evaluated the diagnostic value of the three peptides selected based on Cox regression in distinguishing the AD group from the four non-AD groups (CN, SMC, EMCI, and LMCI). Table 4 illustrates statistically significant differences in the diagnostic performance of CSF Aβ42, tau, P-tau, and the three peptides across the various groups. No significant difference was observed in the accuracy of CSF Aβ42 and VVSSIEQK in distinguishing between the control group and the AD group (*P* = 0.480). In distinguishing between SMC and AD, as well as EMCI and AD, CSF Aβ42 exhibited a higher area under the curve (AUC) than all peptides (*P*-all < 0.05). Nevertheless, there was no significant difference in AUC between AD-related biomarkers and peptides for differentiating between LMCI and AD (*P*-all > 0.05).

**Table 4:** ROC analysis results of cerebrospinal fluid AD biomarkers and peptides for differentiating various diagnoses (*n* = 773)

	Aβ42	*P*-tau	VAELEDEK	VSFELFADK	VVSSIEQK
**AD vs. CN**					
AUC	0.748	0.802	0.629	0.641	0.724
Sensitivity (%)	65.91	81.82	37.88	68.18	75.00
Specificity (%)	79.03	73.91	87.63	57.53	61.83
Threshold value	182.0	24.9	0.0036	0.0083	0.0391
PPV (%)	69.0	69.2	68.5	53.3	58.2
NPV (%)	76.6	85.0	66.5	71.8	77.7
*P* value	< 0.001^***^	< 0.001^***^	< 0.001^***^	< 0.001^***^	< 0.001^***^
**AD vs. SMC**					
AUC	0.967	0.823	0.638	0.705	0.794
Sensitivity (%)	84.85	75.76	37.88	61.36	68.94
Specificity (%)	97.64	81.10	86.61	74.80	84.25
PPV (%)	97.4	80.6	74.6	71.7	82.0
NPV (%)	86.1	76.3	57.3	65.1	72.3
Threshold value	495.4	27.6	0.0036	0.0089	0.0421
*P* value	< 0.001^***^	< 0.001^***^	< 0.001^***^	< 0.001^***^	< 0.001^***^
**AD vs. EMCI**					
AUC	0.824	0.685	0.651	0.587	0.636
Sensitivity (%)	82.58	79.55	56.06	62.88	53.79
Specificity (%)	70.89	60.76	73.42	56.96	72.15
PPV (%)	82.6	77.2	77.9	70.9	76.3
NPV (%)	70.9	64.0	60.0	55.9	58.1
Threshold value	453.4	25.7	0.0044	0.0088	0.0512
*P* value	< 0.001^***^	< 0.001^***^	< 0.001^***^	0.033^*^	< 0.001^***^
**AD vs. LMCI**					
AUC	0.559	0.603	0.562	0.563	0.612
Sensitivity (%)	65.91	75.76	50.76	68.18	57.58
Specificity (%)	47.98	47.79	61.85	47.79	61.45
PPV (%)	40.3	43.5	41.4	40.9	44.2
NPV (%)	72.6	78.8	70.3	73.9	73.2
Threshold value	182.0	27.0	0.0041	0.0083	0.0490
*P* value	0.058	< 0.001^***^	0.045^*^	0.044^*^	< 0.001^***^

Abbreviations: CN, cognitively normal; SMC, subjective memory complaint; EMCI, early mild cognitive impairment; LMCI, late mild cognitive impairment; AD, Alzheimer’s disease; Aβ, β-amyloid; ROC, receiver operator characteristic; AUC, area under the curve; PPV, positive predictive value; NPV, negative predictive value

*P < 0.05; **P < 0.01; ***P < 0.001

Finally, we assessed whether the addition of the three peptides in combination could enhance the accuracy of classical AD biomarkers in diagnosing AD. The combination of Aβ42 and P-tau (AUC = 0.827, 95% confidence interval [CI] = 0.781–0.867, *P* < 0.001, sensitivity 77.27%, and specificity 78.26%) did not surpass the performance of the combination of the three peptides (AUC = 0.872, 95% CI = 0.831–0.907, *P* < 0.001, sensitivity 85.61%, and specificity 76.88%) in differentiating between CN and AD (*P* = 0.084). However, upon adding the three peptides to the combination of Aβ42 and P-tau, the AUC increased to 0.900, significantly higher than the AUC of the combination of Aβ42 and P-tau alone (*P* < 0.001). This suggests that proteomics is comparable to classical AD biomarkers in predicting AD and can even enhance the predictive performance of classical AD biomarkers when combined with proteomics.

### Mediation effect of proteomics on cognition via neuroimaging

**Fig. 3:**
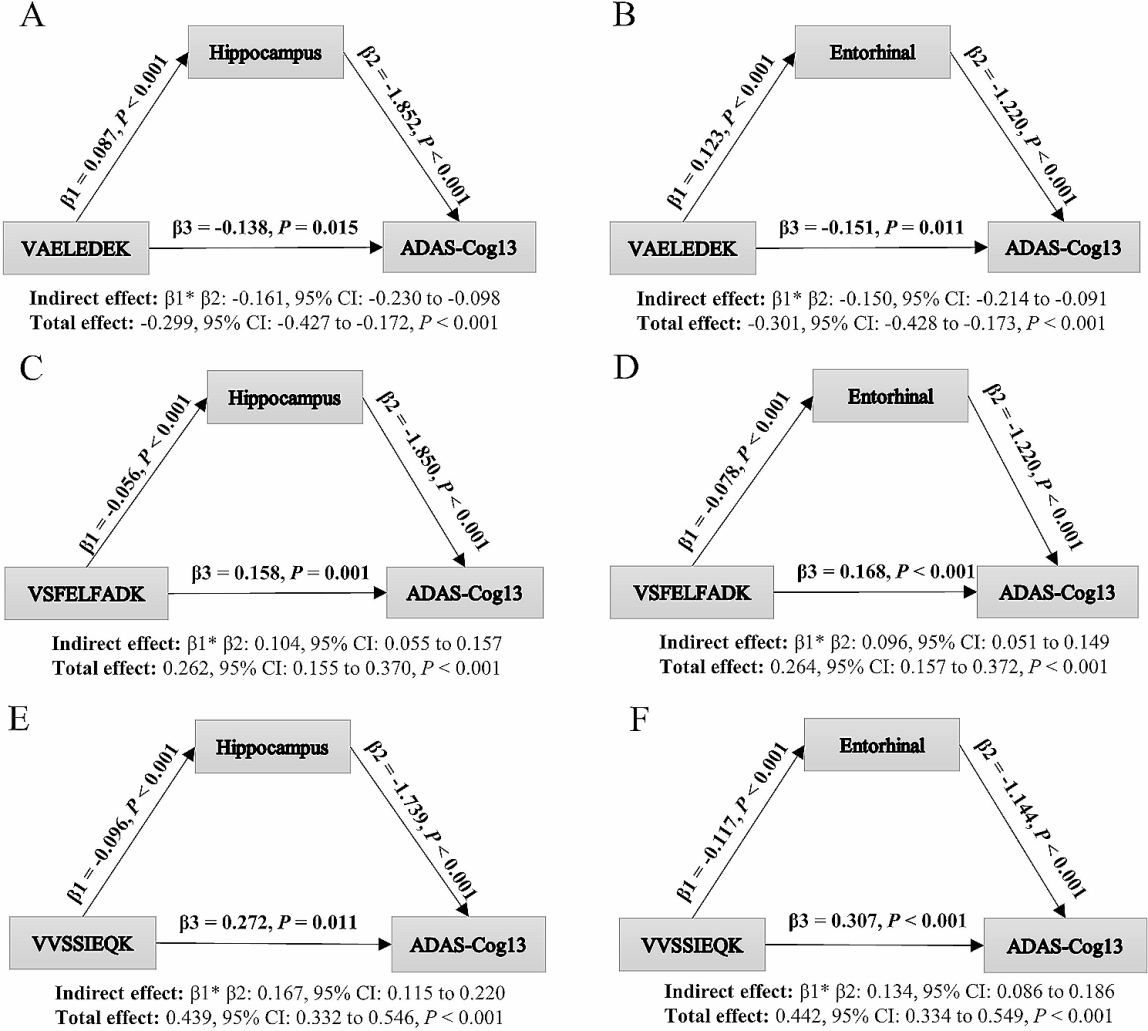
Mediation effects of peptides on cognitive function via hippocampal and entorhinal volumes. **A** VAELEDEK effect on ADAS-Cog 13 mediated by hippocampal volume. **B** VAELEDEK effect on ADAS-Cog 13 mediated by entorhinal volume. **C** VSFELFADK effect on ADAS-Cog 13 mediated by hippocampal volume. **D** VSFELFADK effect on ADAS-Cog 13 mediated by entorhinal volume. **E** VVSSIEQK effect on ADAS-Cog 13 mediated by hippocampal volume. **F** VVSSIEQK effect on ADAS-Cog 13 mediated by entorhinal volume. Abbreviations: ADAS-Cog 13, 13-item cognitive subscale of the Alzheimer’s Disease Assessment Scale

Mediation analysis was employed to examine the indirect effects of peptides on cognitive function (ADAS-Cog 13) through the hippocampus and entorhinal regions (Fig. 3). This analysis focused on three peptides that showed significant differences in the multivariable Cox regression: VAELEDEK, VSFELFADK, and VVSSIEQK. We found that VAELEDEK, VSFELFADK, and VVSSIEQK exhibited mediated effects through the hippocampus with proportions of 53.8%, 39.6%, and 38.0%, respectively. Similarly, VAELEDEK, VSFELFADK, and VVSSIEQK also exhibited mediated effects through the entorhinal region, with proportions of 49.8%, 36.3%, and 30.3%, respectively. Additionally, after adjusting for age, gender, education, and ApoE ε4 status, ADAS-Cog 13 exhibited significant positive correlations with VVSSIEQK, but significant negative correlations with VAELEDEK and VSFELFADK (*P*-all < 0.05).

### Sensitivity analyses

After excluding participants with incomplete data, no significant changes were observed in baseline characteristics (Supplementary Table 2). Furthermore, multivariable Cox regression analysis from the full dataset revealed that Log VAELEDEK, Log VSFELFADK, and Log VVSSIEQK remained significant predictors of conversion to AD (Supplementary Table 3). Additionally, in validation of the selected peptides in Aβ-positive patients following the ATN framework, we observed that both peptides (VAELEDEK and VSFELFADK) remained robust predictors of conversion to AD, while VVSSIEQK did not demonstrate predictive capability for conversion to AD (Supplementary Table 4).

## Discussion

Among the sixty-five candidate biomarkers investigated, three peptides (VAELEDEK, VSFELFADK, and VVSSIEQK) were identified as significant predictors of conversion to AD across the entire AD continuum. Incorporating these three peptides into the predictive model, in combination with Aβ42 and P-tau, improved the accuracy of predicting AD conversion. Furthermore, these three peptides exhibited strong discriminatory power in distinguishing AD from non-AD individuals. Importantly, this study revealed that the impact of these peptides on cognitive function was mediated through changes in hippocampal and entorhinal volumes. In a prior clinical investigation, a linear correlation between Aβ ratios and VAELEDEK and VVSSIEQK was unveiled [17]. Expanding upon these observations, it was ascertained that these two peptides, alongside the recently identified VSFELFADK, harbor promising clinical utility in forecasting the progression to AD.

In this comprehensive proteomics study, we observed a gradual reduction in the number of peptides with differential expression as the disease progressed from CN to AD. This indicates the existence of distinct differences among the five stages of disease progression, which gradually diminish as the disease advances. The observed alterations in protein regulation throughout the course of AD may contribute to the decrease in the number of peptides with differential expression [19]. Different from previous study that used dozens of proteins as a panel to predict AD [20], this study initially screened nine peptides from a pool of sixty-five peptides that exhibited differential expression across the entire AD continuum. These selected peptides have predictive and clinical value for patients at any stage of the disease. Notably, significant changes in forty-seven peptides were observed in individuals with SMC compared to AD. This supports the notion that SMC represents an earlier stage in the continuum of AD progression compared to MCI [21]. Moreover, early pharmacological interventions have shown promise in improving memory and executive function in individuals with SMC [22]. However, the current diagnostic criteria for SMC primarily rely on questionnaire scales and lack objective biochemical markers. Our findings provide novel evidence supporting the efficacy of proteomics in differentiating SMC from AD.

Building upon the findings of the longitudinal study, the peptides VAELEDEK, VSFELFADK, and VVSSIEQK independently demonstrated predictive capabilities for the occurrence of AD conversion as an endpoint event. Furthermore, incorporating these three peptides into the reference model markedly enhances the C-statistic for the conversion to AD. Specifically, these peptides correspond to NPTX2, PPIase A, and 14-3-3 protein zeta/delta, respectively. In a clinical study with a limited sample size, a significant correlation was observed between the rate of NPTX2 changes and cognitive decline [23]. Another study, longitudinally assessing cognitive scores at multiple time points, concluded that baseline NPTX2 levels were linked to cognitive decline [24]. Currently, there is limited clinical research exploring the association between PPIase A, 14-3-3 protein zeta/delta, and AD. A study investigating various isoforms of 14-3-3 protein zeta/delta in the frontal cortex of 12 postmortem AD patients found no significant difference in expression levels compared to control subjects [25]. However, a separate study, measuring multiple 14-3-3 protein isoforms in the cerebrospinal fluid of 52 AD patients, reported that zeta/delta exhibited the highest AUC among all isoforms [26], aligning with our findings. Distinguishing itself from prior studies, this study derived advantages from a larger sample size, recorded AD conversion as a survival outcome in non-AD patients, and adjusted for classical AD-related biomarkers and baseline diagnostic results in a multivariable Cox regression analysis. These methodological enhancements bolstered the reliability of the conclusions. Additionally, the incorporation of proteomics into the AD-related biomarker model augmented the C-statistic, emphasizing the predictive utility of proteomics in evaluating the risk of AD development.

The potential mechanisms underlying the roles of these three CSF biomarkers in AD are as follows. NPTX2, interacting with alpha-amino-3-hydroxy-5-methyl-4-isoxazolepropionic acid-type glutamate receptors, mediates excitatory synapse maturation [27]. Conversely, NPTX1 restricts excitatory synaptic plasticity [28]. Disruption of the dynamic balance between NPTX2 and NPTX1 may lead to impaired synaptic function. Furthermore, C1q participates in the toxicity induced by soluble Aβ oligomers on synapses and long-term potentiation in the hippocampus [29], while reduced expression of NPTX2 activates complement C1q, leading to neurotoxicity, which may be one of the mechanisms contributing to cortical atrophy [30]. PPIase A, previously studied primarily in the context of viruses, belongs to the PPIase family. Cyclophilin A, another member of this family, has been extensively investigated and is known to regulate brain vascular integrity, contributing to neurodegenerative changes [31]. The potential mechanism of PPIase A in AD may be associated with its role in selenium transport to the brain [32]. Impairment of selenium transport may result in increased deposition of amyloid-beta plaques in the brain [33]. While research on the association between PPIase A and AD remains limited, Pin1, as the only known PPIase, has been shown to mediate dendritic spine loss induced by Aβ42 [34]. Both this study and other clinical research [17] indicate a linear correlation between CSF Aβ42 or Aβ ratios and NPTX2, PPIase A, further corroborating previous findings. 14-3-3 proteins, including various isoforms, are highly expressed in the brain, particularly at synapses, and serve as regulatory factors in synaptic transmission and plasticity [35]. They can also co-localize with tau in neurofibrillary tangles, being associated with tau deposition [36], and a clinical study has confirmed their association with cortical atrophy [37]. Although research on the association between 14-3-3 protein zeta/delta and neuronal damage in AD models is lacking, it has been found in a mouse model of spinal muscular atrophy that 14-3-3 protein zeta/delta can activate the MEK/ERK pathway and regulate neuronal survival by interacting with B-Raf [38]. These potential mechanisms offer insights into the potential involvement of these biomarkers in the pathogenesis and progression of AD.

A novel finding of this study was the identification of the mediating role of the hippocampus and entorhinal cortex in the effects of these three proteins on cognitive function, with a moderate mediating effect ranging from 30 to 50%. This suggests that these proteins predominantly exert their biological effects through these specific brain structures. Mediation analysis is a valuable tool used to identify potential variables in causal pathways [39]. By uncovering intermediate variables, it helps elucidate the mechanisms underlying the causal factors. In future studies, interventions targeting these intermediate variables could be explored to influence the outcomes. Previous research utilizing linear mixed models has shown that NPTX2 predicts medial temporal lobe atrophy and decline in memory in AD patients [24]. Our study expands on these findings by presenting a comprehensive causal model. We specifically selected the hippocampus and entorhinal cortex as the mediating brain regions due to their significant impact on cognitive function and their well-established associations with cognition in the field of AD research [4, 40]. This study provides new theoretical evidence supporting interventions targeting these three proteins to potentially impact the hippocampus and entorhinal cortex, thereby potentially slowing down cognitive decline.

This study is notable for including a sufficient number of proteomics samples and conducting a comprehensive analysis of the entire process of AD onset. It provides valuable insights into the potential mechanisms underlying the association between proteomics and AD through longitudinal analyses. However, there are a few limitations that should be acknowledged. Firstly, this study assessed baseline peptides and did not account for the rate of peptide changes, which may also be relevant to AD onset. Secondly, there were some missing values for certain variables. These missing values were handled through deletion during the analysis process, but it is worth noting that the number of missing values was small. Lastly, the follow-up duration in this study was relatively short, spanning only three years. Due to this limitation, we may not have been able to observe longer-term disease progression or effects. Therefore, future studies could consider extending the follow-up period to more comprehensively assess the relevant outcomes. Despite these limitations, the overall results of this study can be considered reliable.

In summary, this study emphasizes that three peptides independently predict the risk of developing AD. The combination of these three peptides with the classical AD biomarker model enhances predictive accuracy. Additionally, these three peptides also demonstrate high accuracy in distinguishing AD from non-AD cases. Finally, we found that the effects of these three peptides on cognitive function are primarily mediated through the volume of the hippocampus and entorhinal cortex. Future research should explore the therapeutic potential of targeting these three peptides to slow down cognitive decline in individuals at risk of developing AD.

### Electronic supplementary material

Below is the link to the electronic supplementary material.

Supplementary Material 1

## Data Availability

Researchers interested in accessing the ADNI data can do so through the LONI Imaging & Data Archive. To apply for access, they can visit the ADNI website at http://adni.loni.usc.edu/data-samples/access-data/.
