# Supplementary material for: Potential prognostic value of CSF-targeted proteomics across the Alzheimer’s disease continuum
Source: BMC Geriatr. 2024 Jun 6;24:501. doi: 10.1186/s12877-024-05104-z (PMC11157758; doi:10.1186/s12877-024-05104-z)
Supplement: Supplementary file 1 — Supplementary Material 1 [file 12877_2024_5104_MOESM1_ESM.docx]

Supplementary Table 1 The numbers (percentages) of participants with missing covariates

| Covariate | Number | Percentage (%) |
| --- | --- | --- |
| Age | 2 | 0.3 |
| Hisp/Latino | 2 | 0.3 |
| ApoE ε4 | 35 | 4.5 |
| CSF Aβ42 | 1 | 0.1 |
| CSF tau | 7 | 0.9 |
| CSF P-tau | 2 | 0.3 |

Abbreviations: ApoE, apolipoprotein E; CSF, cerebrospinal fluid; Aβ, β-amyloid.

Supplementary Table 2 Demographics and clinical characteristics after excluding participants with incomplete covariate data (n = 733)

| Characteristics | CN (n=184) | SMC (n=121) | EMCI (n=66) | LMCI (n=236) | AD (n=126) | *P* value |
| --- | --- | --- | --- | --- | --- | --- |
| Age, years | 73.1 ± 6.2 | 70.9 ± 6.3 | 72.1 ± 7.4 | 73.8 ± 7.7 | 73.9 ± 8.4 | 0.003^**^ |
| Female, n (%) | 106 (57.6) | 81 (66.9) | 29 (43.9) | 80 (33.9) | 50 (39.7) | <0.001^***^ |
| Hisp/Latino, n (%) | 5 (2.7) | 5 (4.1) | 3 (4.5) | 4 (1.7) | 2 (1.6) | 0.418 |
| Education, years | 16 (14 - 18) | 16 (16 - 18) | 16 (13 - 18) | 16 (14 - 18) | 16 (13 - 18) | <0.001^***^ |
| Married status, n (%) | 135 (73.4) | 88 (72.7) | 59 (89.4) | 198 (83.9) | 109 (86.5) | 0.001^**^ |
| ApoE ε4, n (%) | 44 (23.9) | 49 (40.5) | 28 (42.4) | 123 (52.1) | 89 (70.6) | <0.001^***^ |
| CSF Aβ42 (pg/ml) | 260.5 (202.2 - 1140.2) | 1117.0 (831.6 - 1679.5) | 736.2 (224.5 - 1375.7) | 163.0 (131.0 -270.2) | 146.5 (128.7 - 276.7) | <0.001^***^ |
| CSF tau (pg/ml) | 91.5 (58.0 - 183.6) | 220.5 (171.6- 282.5) | 208.6 (108.9 - 280.7) | 102.0 (68.5 - 163.5) | 137.0 (89.0 - 215.5) | <0.001^***^ |
| CSF P-tau (pg/ml) | 19.1 (15.0 - 25.0) | 19.2 (14.9 - 25.9) | 22.5 (17.2 - 41.0) | 30.0 (19.0 - 43.0) | 35.0 (27.9 - 48.4) | <0.001^***^ |

Note: Values are expressed as mean ± SD, median (interquartile range), or frequency (%).

Abbreviations: CN, cognitively normal; SMC, subjective memory complaint; EMCI, early mild cognitive impairment; LMCI, late mild cognitive impairment; AD, Alzheimer's disease; ApoE, apolipoprotein E; CSF, cerebrospinal fluid; Aβ, β-amyloid.

**P* < 0.05; ***P* < 0.01; ****P* < 0.001

Supplementary Table 3 Univariable and multivariable Cox regression analysis for conversion to AD as the outcome measure in non-AD patients after excluding participants with incomplete covariate data (n = 550)

| Parameter | Univariable analysis  HR (95% CI) | *P* value | Multivariable analysis  HR (95% CI) | *P* value |
| --- | --- | --- | --- | --- |
| Age | 1.02 (1.00 - 1.05) | 0.076 |  |  |
| Female | 0.71 (0.51 - 1.00) | 0.049^*^ | 1.41 (0.95 - 2.10) | 0.092 |
| Education | 0.95 (0.89 - 1.00) | 0.056 |  |  |
| Married status | 0.50 (0.30 - 0.84) | 0.008^**^ | 0.71 (0.40 - 1.25) | 0.239 |
| Hisp/Latino | 0.59 (0.15 - 2.40) | 0.462 |  |  |
| ApoE ε4 | 2.64 (1.89 - 3.70) | <0.001^***^ | 1.00 (0.67 - 1.48) | 0.996 |
| Baseline diagnosis |  |  |  |  |
| CN | Reference |  |  |  |
| SMC | 0.21 (0.03 - 1.62) | 0.135 | 1.31 (0.15 - 11.17) | 0.807 |
| EMCI | 3.72 (1.71 - 8.09) | 0.001^***^ | 5.16 (2.25 - 11.85) | <0.001^***^ |
| LMCI | 13.31 (7.55 - 23.46) | <0.001^***^ | 10.15 (5.36 - 19.22) | <0.001^***^ |
| **AD biomarkers** |  |  |  |  |
| Log Aβ42 | 0.02 (0.01 - 0.06) | <0.001^***^ | 0.20 (0.08 - 0.51) | 0.001^***^ |
| Log Tau | 1.10 (0.62 - 1.97) | 0.746 |  |  |
| Log P-tau | 41.39 (19.45 - 88.08) | <0.001^***^ | 4.75 (1.62 - 13.91) | 0.004^**^ |
| **Underexpressed peptides** |  |  |  |  |
| Log ADQDTIR | 0.17 (0.07 - 0.39) | <0.001^***^ | 0.21 (0.02 - 2.50) | 0.215 |
| Log EPVAGDAVPGPK | 0.24 (0.11 - 0.49) | <0.001^***^ | 0.30 (0.01 - 14.44) | 0.540 |
| Log GLQEAAEER | 0.21 (0.10 - 0.45) | <0.001^***^ | 0.53 (0.01 - 24.87) | 0.748 |
| Log VAELEDEK | 0.12 (0.04 - 0.33) | <0.001^***^ | 0.04 (0.00 - 0.37) | 0.004^**^ |
| **Overexpressed peptides** |  |  |  |  |
| Log LNVTEQEK | 5.38 (1.92 - 15.09) | 0.001^**^ | 5.07 (0.47 - 54.46) | 0.180 |
| Log NLLSVAYK | 7.24 (3.15 - 16.64) | <0.001^***^ | 10.45 (0.13 - 837.04) | 0.294 |
| Log VISSIEQK | 7.84 (3.05 - 20.20) | <0.001^***^ | 6.64 (0.08 - 563.04) | 0.403 |
| Log VSFELFADK | 3.57 (1.54 - 8.31) | 0.003^**^ | 0.01 (0.00 - 0.09) | <0.001^***^ |
| Log VVSSIEQK | 9.61 (4.32 - 21.34) | <0.001^***^ | 40.01 (2.67 - 600.30) | 0.008^**^ |

Note: AD cerebrospinal fluid biomarkers and peptides were normalized by log_10_ transformation.

Abbreviations: CN, cognitively normal; SMC, subjective memory complaint; EMCI, early mild cognitive impairment; LMCI, late mild cognitive impairment; AD, Alzheimer's disease; Aβ, β-amyloid; HR, hazard ratios; CI, confidence interval.

**P* < 0.05; ***P* < 0.01; ****P* < 0.001.

Supplementary Table 4 Univariable and multivariable Cox regression analysis for conversion to AD as the outcome measure in β-amyloid positive non-AD patients (n = 431)

| Parameter | Univariable analysis  HR (95% CI) | *P* value | Multivariable analysis  HR (95% CI) | *P* value |
| --- | --- | --- | --- | --- |
| Age | 1.01 (0.98 - 1.03) | 0.716 |  |  |
| Female | 0.79 (0.56 - 1.10) | 0.159 |  |  |
| Education | 0.97 (0.92 - 1.03) | 0.360 |  |  |
| Married status | 0.50 (0.30 - 0.85) | 0.010^**^ | 0.72 (0.42 - 1.25) | 0.249 |
| Hisp/Latino | 0.68 (0.17 - 2.77) | 0.595 |  |  |
| ApoE ε4 | 2.18 (1.55 - 3.05) | <0.001^***^ | 0.88 (0.60 - 1.28) | 0.493 |
| Baseline diagnosis |  |  |  |  |
| CN | Reference |  |  |  |
| SMC | 0.44 (0.06 - 3.39) | 0.431 | 1.00 (0.12 - 8.37) | 0.999 |
| EMCI | 3.74 (1.69 - 8.29) | 0.001^***^ | 4.09 (1.73 - 9.64) | 0.001^**^ |
| LMCI | 11.88 (6.72 - 21.01) | <0.001^***^ | 8.05 (4.23 - 15.30) | <0.001^***^ |
| **AD biomarkers** |  |  |  |  |
| Log Aβ42 | 0.03 (0.01 - 0.08) | <0.001^***^ | 0.05(0.01 - 0.17) | <0.001^***^ |
| Log Tau | 2.49 (1.43 - 4.32) | 0.001^**^ | 15.14(4.33 - 52.92) | <0.001^***^ |
| Log P-tau | 22.71 (10.70 - 48.20) | <0.001^***^ | 0.74 (0.17 - 3.19) | 0.683 |
| **Underexpressed peptides** |  |  |  |  |
| Log ADQDTIR | 0.25 (0.11 - 0.57) | <0.001^***^ | 0.09 (0.01 - 1.19) | 0.068 |
| Log EPVAGDAVPGPK | 0.31 (0.15 - 0.62) | 0.001^**^ | 0.07 (0.00 - 3.44) | 0.182 |
| Log GLQEAAEER | 0.30 (0.15 - 0.62) | 0.001^**^ | 1.78 (0.04 - 85.63) | 0.770 |
| Log VAELEDEK | 0.17 (0.06 - 0.46) | <0.001^***^ | 0.07 (0.01 - 0.60) | 0.015^*^ |
| **Overexpressed peptides** |  |  |  |  |
| Log LNVTEQEK | 4.08 (1.46 - 11.45) | 0.008^**^ | 6.12 (0.59 - 64.06) | 0.130 |
| Log NLLSVAYK | 5.12 (2.21 - 11.89) | <0.001^***^ | 20.05 (0.26 - 1536.00) | 0.176 |
| Log VISSIEQK | 5.66 (2.18 - 14.73) | <0.001^***^ | 3.34 (0.04 - 284.20) | 0.595 |
| Log VSFELFADK | 2.93 (1.26 - 6.80) | 0.013^*^ | 0.03 (0.00 - 0.36) | 0.005^***^ |
| Log VVSSIEQK | 7.15 (3.11 - 16.44) | <0.001^***^ | 12.63 (0.73 - 217.63) | 0.081 |

Note: AD cerebrospinal fluid biomarkers and peptides were normalized by log_10_ transformation.

Abbreviations: CN, cognitively normal; SMC, subjective memory complaint; EMCI, early mild cognitive impairment; LMCI, late mild cognitive impairment; AD, Alzheimer's disease; Aβ, β-amyloid; HR, hazard ratios; CI, confidence interval.

**P* < 0.05; ***P* < 0.01; ****P* < 0.001.
